# Act in time: primary health care professionals’, internal facilitators’, and managers’ experiences of working health-promotively after a 12-month implementation intervention: a qualitative study using normalization process theory

**DOI:** 10.1186/s12875-026-03181-0

**Published:** 2026-01-22

**Authors:** Berntsson Karin, Nilsagård Ylva, Hälleberg-Nyman Maria, Wallin Lars, Nilsing Strid Emma

**Affiliations:** 1 University Healthcare Research Centre, Faculty of Medicine and Health, Post Graduate School for Integrated Care, Örebro University, Örebro, SE-701 85 Sweden; 2 Department of Health and Welfare, Dalarna University, Falun, SE-791 88 Sweden

**Keywords:** Health promotion, Implementation science, Primary health care, Qualitative research

## Abstract

**Background:**

Healthy-lifestyle-promoting practices are recommended to reduce the prevalence of non-communicable diseases and increase health, but are underutilized in Swedish primary health care (PHC). As part of the Act in Time project, a 12-month multifaceted implementation intervention to support the uptake of a clinical intervention offering lifestyle-screening forms and counselling to patients with planned visits, was evaluated in a PHC setting. This study aimed to explore the experiences of PHC professionals, internal facilitators, and managers working with health promotion after receiving the 12-month implementation intervention.

**Methods:**

A qualitative study was conducted at five PHC units in Sweden using interviews with managers (*n* = 9) and internal facilitators (*n* = 10) and focus group discussions (*n* = 5) with physicians, nurses, counsellors, and physiotherapists (*n* = 18). The data were analysed with qualitative content analysis, first inductively and then deductively by mapping the data against the 12 constructs of Normalization Process Theory.

**Results:**

Implementation of the health-promoting practice was affected by contextual factors such as attitudes and available resources. The group dynamics at the PHC centres and the managers’ role as leaders were important for finding solutions to enact the health-promotion practice.

Health-promotion practice was seen as a natural development of PHC. A common focus and opportunity to influence created a sense of coherence. Feelings of autonomy enabled the professionals to collaborate and strengthened participation in the implementation intervention. The internal facilitators helped to guide the PHC centres forward in the implementation process and created strategies to integrate the health-promotion practice into existing clinical practice.

Uptake of the clinical intervention led to a more structural and holistic way of working with lifestyle habits, with patients taking an active part. The professionals’ competencies became more visible by working together, but frustration was also expressed due to different levels of engagement in the health-promotion practice.

**Conclusions:**

Health-promotion practice can be normalized as routine work in PHC with targeted support but requires tailored strategies that rely on existing group dynamics and the manager’s role. To create motivation for providing health promotion, inter-professional collaboration is a key factor that ensures shared ownership of the implementation intervention.

**Trial registration:**

This study is part of the Act in Time project, registered at ClinicalTrials.gov on 4 March 2021 (ref NCT04 799860).

**Supplementary Information:**

The online version contains supplementary material available at 10.1186/s12875-026-03181-0.

## Background

Primary health care (PHC) is intended as the first contact for all people seeking non-emergency care, and has the goal of providing continuous, comprehensive, and coordinated person-centred care. PHC should have a holistic approach, with a strong emphasis on health promotion [1]. Unhealthy lifestyle behaviours such as physical inactivity, unhealthy eating habits, harmful use of alcohol, and tobacco use are important and modifiable risk factors for the onset and progression of common non-communicable diseases that cause death and illness [2, 3]. Most people worldwide have at least one unhealthy lifestyle habit, and it is common to have multiple unhealthy habits at the same time [4, 5].

According to the World Health Organization, all countries would benefit from finding strategies to counter the impact of non-communicable diseases on health [6]. However as there is no one-size-fits-all action plan, national contexts need to be taken into account to address the challenge of unhealthy lifestyles. Lifestyle interventions are recommended as a cost-effective treatment in PHC, based on findings of low costs and improved health [7, 8]. Several countries, including Sweden, have guidelines with recommendations for the prevention of non-communicable diseases by promoting healthy lifestyles [9]. In parts of the Swedish PHC context, targeted health dialogues have been used to promote healthy lifestyles [10], however simple advice and consulting conversations are still underutilized in PHC [11]. In 2023, only about 6–7% of PHC patients in Sweden received advice on physical activity and as few as 1% received advice on alcohol habits [12]. PHC seems not to be the first option for patients seeking lifestyle advice, perhaps partially due to a lack of awareness that such guidance is available there [13]. This is a concern, as previous studies have shown that cardiovascular events and deaths were significantly reduced in patients who received structural lifestyle advice in PHC [10, 14, 15]. Time constraints and limited counselling knowledge have been reported as the main barriers to health promotion, whereas a positive attitude and recognition of its relevance to patient health were key facilitators [16].

Among patients who perceive a need for lifestyle changes, the proportion who want support from PHC is generally above 80% in both Sweden and the United States [17]. The results from a European study highlighted that patients expect questions about lifestyle habits from PHC professionals, and are more likely to experience a lack of engagement if not asked [18]. Lifestyle advice and counselling may lead to reflection and awareness among patients about their own health [19]. However, for health promotion to be integrated into PHC practice, PHC professionals need to have the ability to support their patients in changing to healthier lifestyle habits. As previously highlighted, PHC professionals have a positive view of health promotion as well as a desire to become involved, which demonstrates a new way of thinking about providing health care [11]. This offers the potential to develop a greater focus on health promotion [20].

**Fig. 1:**
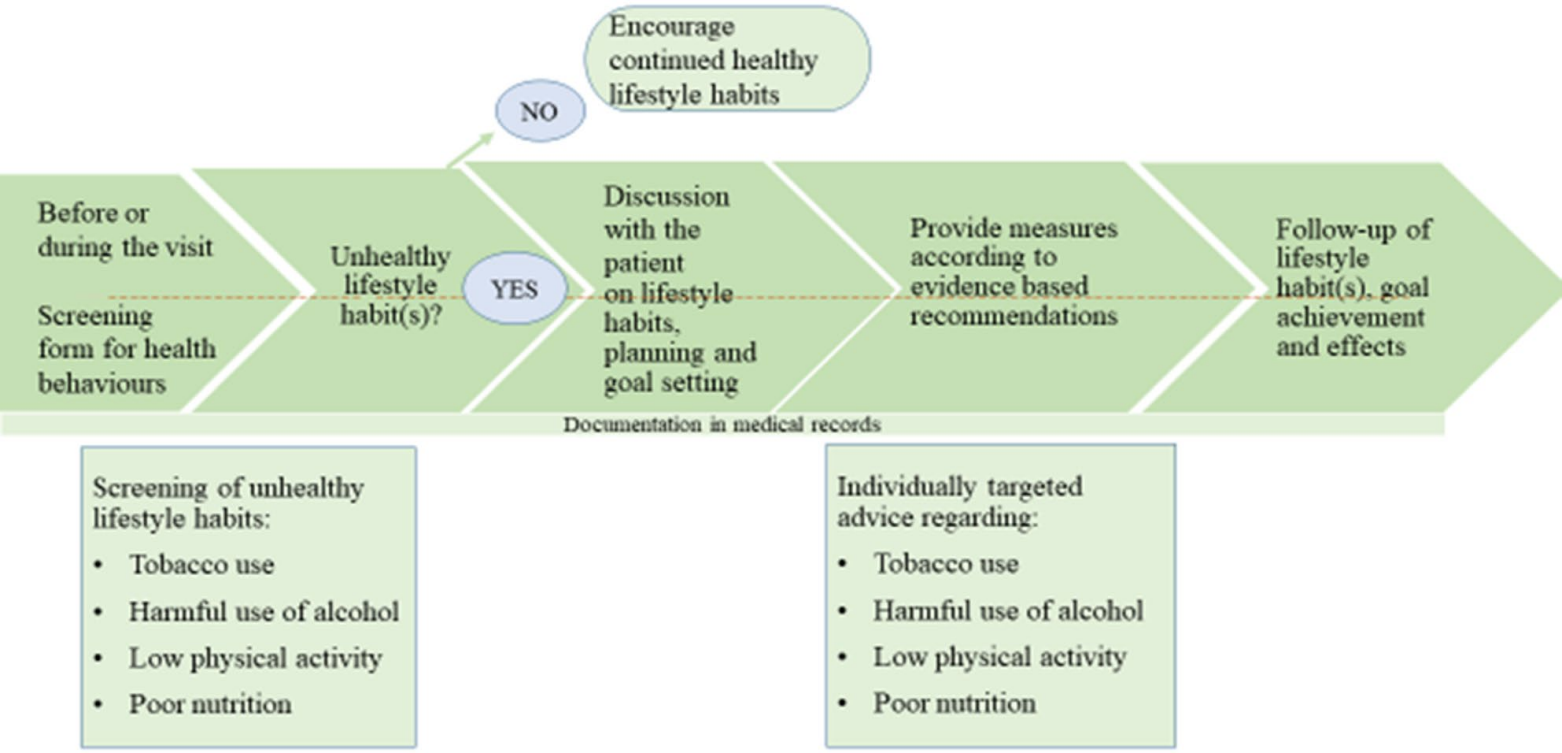
Overview of the clinical intervention as described in a previously-published study protocol [21]

The present study is part of the Act in Time project, which aims to provide a more generalizable understanding of the implementation of a clinical intervention enabling a health-promoting practice in a PHC context [21]. All PHC professionals encountering patients at their centre were asked to encourage the patients to complete a lifestyle screening form in advance (Supplementary File 1), to invite them to a dialogue about potentially unhealthy lifestyles, and to register health-promoting actions in the medical record using the Swedish classification of health intervention codes (Fig. 1). The goal was to offer lifestyle advice to all patients with planned visits and health conditions that would benefit from this. However, the PHC centres were able to decide the order in which to target different patient groups.

The dialogue about lifestyles was based on the Swedish national guidelines for health promotion and disease prevention [22]. To implement new ways of working with health promotion, more knowledge is needed about successful implementation interventions [23]. The implementation intervention in the Act in Time project, described in detail in a study protocol [21], included multifaceted strategies with both external and internal facilitators (EFs/IFs) [24], support to managers, and the steps and education from the Astrakan model of leading change [25]. The use of an established theory provides tools to understand what affects the adoption and sustainability of an implementation intervention [26]; for this purpose, the present study used Normalization Process Theory (NPT). NPT describes the nature of implementation as a social process of collective actions and social mechanisms to explain the different parts of the implementation process [27]. An intervention is considered implemented when it becomes a natural part of daily work. NPT consists of 12 constructs that are divided into three domains: implementation context, implementation mechanisms, and implementation outcomes [28] (Supplementary File 2).

Previous research about working health-promotively in PHC has focused on physicians’ and nurses’ perceptions and use of available resources and tools [20, 29–31], but without the use of a structured implementation intervention as foundation. The effectiveness of implementing health-promotion interventions and the experiences of different PHC professionals in working health-promotively therefore still needs to be explored in the light of implementation science. A qualitative study during the pre-implementation phase for the Act in Time project found that PHC managers, IFs, and PHC professionals believed that strong leadership support from both senior and PHC managers was vital for prioritizing health promotion. This enabled PHC centres to build capacity and take control of the implementation intervention [32]. PHC professionals valued health promotion, as it was believed to meet patients’ needs and promote cooperation, but there was a need for the implementation intervention to address barriers to incorporating health promotion into existing work [33]. These findings were taken into consideration when refining a structured and multifaceted implementation intervention aimed at achieving a more health-promotive practice in PHC. This intervention was based on the Astrakan model for leading change, using EFs and IFs and stressing a combined top-down and bottom-up perspective. A non-randomized parallel group study evaluating the adoption of the intervention found that during the implementation phase, the intervention centres sent out 8.6 times as many screening forms as the control centres. Moreover, in comparison to controls, the intervention group showed a statistically significantly higher relative uptake rate of health-promotion activities that was sustained through the post-intervention phase [34]. However, further knowledge is needed to understand and evaluate how health promotion may become routine in a PHC setting. The present study aimed to explore the experiences of PHC professionals, IFs, and managers working with health promotion after a 12-month implementation intervention, in order to contribute new knowledge that may be important when implementing health-promotion practice in a PHC context.

## Methods

### Study design and setting

This study had a qualitative explorative and descriptive design, and used individual interviews and focus group discussions (FGDs) [35] conducted within PHC in a Swedish region with PHC centres serving approximately 307 000 inhabitants. All the PHC centres in the region were invited to participate in the Act in Time project and given information about the multifaceted implementation intervention that was planned to support a health-promotive practice in PHC. Five centres volunteered to participate, and five other centres served as matched controls [20]. These centres represented rural, metropolitan, and medium-sized urban locations of varying socioeconomic status, and were thus considered representative of PHC in Sweden. The implementation intervention was directed to all PHC professionals with treatment responsibility for patients at the intervention centres. All participating intervention centres received a small financial compensation (60 000 SEK) after completing the steps of the implementation process.

The participating PHC centres received 12 months of implementation support from trained EFs [21]. At each centre, two professionals were appointed by their manager to take on the role of IF, with support from two EFs. The EFs comprised four employees at the Region Örebro development unit who had expert knowledge of working with lifestyle habits as well as knowledge of change management. The PHC centres had the opportunity to tailor the implementation intervention to fit into the existing context with guidance from the EFs; for example, when prioritising target groups and choosing methods to arouse interest in working with health-promotive activities. Since the pre-implementation study revealed that PHC professionals require support to prioritize health-promotive work [32], the experiences of managers regarding their role in the implementation intervention were collected. The implementation intervention was á priori designed to promote sustainability and encourage further development.

### Participants

After the end of the 12-month implementation intervention, various personnel from the five participating PHC centres were invited to participate in interviews and FGDs: senior managers (i.e. managers at the higher organizational level), managers at PHC centres, IFs, and a sample of PHC professionals. The invitation included information about the purpose and procedures of the study. All persons invited to individual interviews agreed to participate and provided their informed consent. In total, three senior managers, six PHC managers, and ten IFs were interviewed. At each PHC centre, the manager or one of the appointed IFs forwarded the invitation to participate in a FGD to the PHC professionals at their centre. Those selected for the FGD agreed to participate, and provided their informed consent before the FGD began. We sought to include a diversity of professionals in each group; for example, counsellors, general practitioners, nurses, and physiotherapists.

### Data collection

Semi-structured interview guides based on NPT were developed by the research group for the individual interviews and FGDs (Supplementary File 3). The questions focused on what it was like to work health-promotively, and what the informants did to integrate health promotion into the existing workflow. The questions were open ended to encourage the informants to talk freely. Data collection took place four months after the 12-month implementation intervention, between February and August 2023, depending on when the PHC centre started the intervention. Interviews were conducted at the PHC centres or by video conference (Visiba Care). All interviews and FGDs were conducted by one of the authors (ENS), with another author (KB) assisting at the FGDs by taking field notes and observing the interactions. All interviews and FGDs were audio recorded and transcribed verbatim by a professional transcriber. The transcripts were checked against the audio files for consistency and then imported into version 14 of NVivo software (Lumivero, 2023. https://lumivero.com/products/nvivo/) for managing and coding of data.

### Data analysis

A qualitative inductive-deductive content analysis was used, in a structured and stepwise process with a manifest interpretation level. This began with repeated reading of the transcribed data and listening to audio files to capture tone and unspoken messages and to gain a deeper understanding of the data and a sense of the whole. All transcribed text that corresponded to the aim was extracted. The data were condensed and initially coded inductively to discover similarities and dissimilarities. This inductive phase was guided by Elo and Kyngäs [36] and processed using NVivo. The data from senior managers, managers, and PHC professionals were coded and held separately during the entire data analysis. Coded data were grouped by content, and different colours were used to make the initial coding of the data more visible.

The analytical process then continued with a deductive approach. A structured analysis matrix was developed, conceptually based on the domains and constructs of NPT [28] and with empirically grounded keywords from the initial inductive analysis. The codes that came from the inductive analysis were used as keywords in the matrix (Supplementary File 2). With help from the matrix, the coded data were mapped into the NPT domains and constructs [28]. The deductive analysis process is exemplified in Table 1. Only data that fitted the NPT coding map were categorized.

**Table 1:** Example of the data analysis process

Quotation	Code	NPT construct (subcategories)	NPT domain (main categories)
The different professional groups have made different progress, but the conditions in the different groups are quite different, which affects how far and how much progress has been made (IF 1).	The professions are at different development stages with different conditions influencing progress.	Adaptive execution	Implementation context
That I don’t in any way assume things about the patient because I ask these questions, but that this is a kind of a base that we start from because you’ve filled this in and most people fill it in when they come here (IF 2).	I don’t make assumptions about the patient; these questions are just a starting point.	Collective actions	Implementation mechanisms
The feeling that, that, well, the conversation has changed, and that it’s more, well, my colleagues can steer the conversation based on their experience, because now they have experience of it (IF 1).	The conversation has changed, and now my colleagues can guide the conversation based on their experiences	Normative restructuring	Implementation outcomes

The first author (KB) was mainly responsible for the coding of the data, but this was checked and discussed regularly with ENS. Continuous discussions were held in the research group with KB, ENS, YN, and MHN through every step of the analysis process. Disagreements were solved in consensus discussions. In this way, the findings that answered the research question were based on the researchers’ analysis. Quotations were used to illustrate the interpretation of the data [37].

### Reflexivity

KB is a registered district nurse and PhD student. ENS is a registered physiotherapist with a PhD and experience of qualitative research and implementation science. YN is a registered physiotherapist, senior researcher, and certified change leader with experience in implementation science. MHN is a registered nurse with a PhD, formal training in qualitative content analysis, and a special interest in implementation science. LW is a registered nurse and internationally reputed senior researcher within implementation science. YN, ENS, LW, and MHN are all experienced in using qualitative methods. All but one of the researchers (LW) are female. The Act in Time project was planned by YN, ENS, and LW, and is led by YN. These authors had no direct contact with the professionals at the PHCs. LW plays an advisory role in the project. The researchers conducting the interviews and FGDs had no prior relationships with the informants. KB, who was not involved in the implementation intervention, independently identified the meaning units from the unit of analysis. The three senior managers were part of the advisory board for the Act in Time project.

## Results

In total, 9 managers and 10 IFs participated in interviews, and 18 PHC professionals participated in 5 FGDs (Table 2).

**Table 2:** Characteristics of participants in the interviews and focus group discussions (FGDs)

	Individual interviews	Individual interviews	FGD A	FGD B	FGD C	FGD D	FGD E
	Managers ^a^	IFs	Staff	Staff	Staff	Staff	Staff
Included respondents, n	9	10	4	4	3	3	4
Length of interview in minutes, mean (range)	36 (31–48)	50 (33–77)	46	45	44	52	48
Sex, n							
Female	8	8	3	3	1	3	3
Male	1	2	1	1	2	0	1
Profession, n							
Licenced practice nurse	-	1	1	0	0	1	1
District nurse	-	2	1	1	0	0	1
Assistant nurse	-	0	0	0	0	1	0
Physiotherapist	-	4	1	1	1	0	0
Physician	-	0	0	0	1	0	1
Midwife	-	1	1	1	0	0	0
Counsellor	-	1	0	1	1	1	1
Rehabilitation coordinator	-	1	0	0	0	0	0
Experience in primary care, years, mean (range)	-	12.2 (2–35)	6.25 (5–7)	4.75 (1–8)	6 (3–20)	2.6 (1–5)	4.25 (1–7)

^a^ Primary health care managers and senior managers. Information on profession and years of experience in primary care was missing for these participants. IF = internal facilitator

The PHC professionals’, IFs’, and managers’ experiences of health-promoting practice are presented below in terms of the three domains of NPT: implementation context, implementation mechanism, and implementation outcome. The 12 constructs of NPT (Supplementary File 2) are used as sub headlines.

### Implementation context

The implementation context describes the environment at the PHC centres and the influence that internal and external factors had on the implementation of the health-promotive practice. Senior managers shared insights under the context domain, but were less involved in implementation mechanisms and outcomes as they did not have clinical patient contacts.

### Strategic intentions

External and internal factors influenced the PHC centres’ environment and shaped the planning of the health-promotively practice. The external factors included research about the impact of lifestyle on health, and the current social situation with an ageing population and limited resources.

*We [the PHC] won’t be able to cope with the burden of more old people and fewer care workers in the future; we need to ensure a healthier population. (IF 7)*

National and political decisions on health promotion as part of the PHC’s mission comprised another external factor, which created internal factors of awareness and acceptance of the health promotion task. In the past, health promotion was considered a low or non-priority issue, as acute care was prioritized with no room for anything else, leading to frustration. The professionals requested more guidance and leadership from their managers on how to prioritize health promotion in relation to acute care. Managers expressed the desire for a trust-based model including frameworks without micromanagement and space for PHC professionals to find their own prioritization strategies and hence shape the implementation context.

*We want a trust-based model where we provide the framework but don’t micromanage*,* leaving room for creativity and individual solutions. (Manager 3)*

### Adaptive execution

The strategic intentions created opportunities for adaptive execution of the health-promoting practice. Despite the described obstacles stemming from prioritization of the broad mission of PHC, high workload, demands of high telephone availability, and increased administrative burden, the majority of the PHC professionals perceived the health-promoting practice as relevant. This enabled them to adapt and make the intervention workable. Competencies in the working group became visible during the implementation intervention and were helpful both for the adaptive execution of the health-promotive practice and for developing strategies.

*Who are the people [the PHC professionals] in front of me [as a manager]? What are we going to do? Is it a temporary thing that will be over in a week? Is it something that is going to last for years? I know my people; I know they have skills. (Manager 6)*

An appropriately sized team of permanent PHC professionals was thought to facilitate a successful implementation intervention according to IF. However, the PHC centres’ context was described as an understaffed and fast-paced working environment where working conditions could vary between different professions; this required tailored strategies. The relationship between the PHC manager and the PHC professionals was emphasized as important for adaptive execution. When PHC professionals communicated needs for training to fulfil the health-promotion task, it was important for them to get support from their PHC manager.

### Negotiating capability

There was an initial concern about intruding, as asking patients questions about their lifestyle habits was considered to touch on sensitive topics. This created an uncertainty that the PHC professional needed to overcome. However, they described that health promotion often fitted well into their existing ways of working when addressed in the right situations. Patients seeking PHC due to asthma/COPD, mental health problems, and newly diagnosed hypertension were examples of appropriate occasions when it became natural to discuss lifestyle habits. Wax plugs, PICC line diversions, or certain visits for foot or knee problems were given as examples of less-appropriate occasions. The initial concern among the PHC professionals that patients would object to discussing lifestyle habits turned out to be mostly unwarranted.

*It has happened that the patient has said no [to talking about health promotion]*,* I’m here for my knee*,* but that happened in about two out of 500 visits*,* so it’s very rare that anyone asks or wonders why. (IF 6)*

### Reframing organizational logic

The social structures at the PHC centres, such as group dynamics, were emphasized as affecting the possibility of implementing the health-promotion practice.

*I think the group dynamics are important in a change. Some things are more prioritized than others depending on who’s working*,* who’s interested. (IF 9)*

Well-functioning group dynamics were described by IF as a healthy and stable group of PHC professionals, appreciating the new way of working and showing inner motivation. This shaped a positive implementation environment. The inner motivation was affected by the professionals’ attitudes towards health-promotive work, and formed by their own lifestyle habits and beliefs about the likelihood of changing the habits of adults. Some professionals were described by managers as more receptive to change than others, and some professional workgroups could be dominated by negative or positive attitudes. Thus, it was challenging to involve everyone at the PHC centres in the implementation of the health-promotion practice. According to IF, in some cases, the diverse attitudes led to tensions in the working groups between those who wanted to work with health promotion and those who did not.

### Implementation mechanisms

The domain of implementation mechanisms describes the purposive social actions and collaborative work that took place at the PHCs, involving the investment of personal and group resources to achieve the goal of integrating the health-promoting practice into ordinary work.

### Coherence-building

Managers said that it was due time to increase health-promotion work, and so they welcomed being part of a process that required a collective change involving all professionals at the PHC centre. PHC professionals who were used to addressing lifestyle habits in patient contacts expressed that the health-promoting practice provided more structure to their work. Others described insufficient knowledge or training in health promotion, leading to uncertainty in how to address lifestyle habits.

The informants expressed that the health-promotion practice required inter-professional collaboration, because health promotion was seen as a shared responsibility that required a mutual approach for the entire PHC centre. The professionals at each PHC centre had come together to agree on what they could offer. A goal was set to integrate health-promoting practice into ordinary work, including being prepared to provide lifestyle advice when lifestyle habits could affect health.

*We would benefit if people took care of their health earlier in life*,* because then there would be fewer people seeking primary care. (FGD 5:1)*

*You can spark a thought or a desire for lifestyle change*,* but it’s hard work for the patients; they’re the ones who must do the work*,* we can only be there to support them*,* and some of them need a lot of support. (FGD 5:2)*

### Cognitive participation

An appointed leader was considered necessary for the initiation of a successful implementation and to lead the way forward, and the IFs were used as this key person. The IFs described an ongoing communication with everyone at the PHC centres. However, there were some criticisms from the PHC professionals that the health-promotion practice had not been sufficiently introduced to all professional groups. Some personnel did not participate in this intervention at all, while others participated fully in the efforts to include health-promotion practice in the centre’s routine work. Attitudes differed between the various health centres; at different centres, negativity and positivity came from different professional groups.

Managers and IFs did not want to interfere with how the PHC professionals organized their patient visits; instead, they promoted autonomy in how to incorporate the health-promotion practice into ordinary work. There was a desire to support feelings of inner motivation, with the hope that health-promotion practice would be considered enjoyable and beneficial work rather than merely a requirement.

*It [the health-promotion practice] shouldn’t be forced*,* it should be fun*,* and you should see the effect and benefit*,* both for yourself and for the patient’s health. (IF 4)*

### Collective actions

The PHC professionals acted together to ensure equal care for their patients. All PHC professionals with patient contacts at the PHC centre had the same authority to address lifestyle when needed, and there was an intention that every PHC professional should be able to provide simple lifestyle advice. Planting an idea and cultivating it with repeated questions about lifestyle habits was described by both IF and managers as an effective long-term strategy. Lifestyle counselling strategies were intended to avoid infringing on the patient’s privacy, and instead to ensure a respectful approach that considered patient perspectives.

It was seen as important to have a well-developed plan for following up the lifestyle screening form, as without follow up, the patient’s trust could be damaged. Concerns were expressed about how to support patients who needed qualified counselling about lifestyle changes, due to limited resources at the PHC centres. One suggested solution was the appointment of coordinators who were experts in lifestyle behaviours and who could facilitate the embedding of the health-promoting practice.

*It’s good to create routines and very clearly summarize how to deal with health-promotion problems; who to refer to*,* and how to raise problems with patients. (Manager 4)*

### Reflexive monitoring

Managers and IFs emphasized the importance of feedback to identify barriers to health-promotion activities. They considered changes in statistics, such as the number of submitted lifestyle screening forms and registered health-promoting activities, to be a valuable means of evaluating progress and sustaining motivation. The PHC professionals’ collective and individual judgements about the value of health-promotion practice were influenced by their patients’ reactions and responses. For example, the PHC professionals mentioned how much they enjoyed asking patients about tobacco use because the response was often positive, with a reflective patient who wanted help to quit. They had found that the patients appeared to be satisfied and acknowledged, further increasing their desire to work more health-promotively and fostering a greater focus on health promotion.

*A physician gave feedback on a patient who had changed [lifestyle] after a month; [the physician said] I need to do more of this [health promotion] because the values have gone down.* (Manager 3)

### Implementation outcomes

The implementation outcome domain shows the effect of the implementation mechanisms; that is, how the practice at the PHC centres had changed when the health-promoting practice was implemented.

### Intervention performance

The PHC professionals who had adopted the health-promotive way of working described an increased focus and awareness of the impact of lifestyle factors, resulting in all lifestyle behaviours being addressed with a more holistic view of the patients. Patients were perceived to be more involved in their own care than before. This was viewed as a co-operation with the patient, where agreement about treatment led to increased motivation for the PHC professional to address healthy lifestyles. It was not always necessary to completely change the way of working, but rather to adapt and develop existing practices.

*Part of the process has been to realize that the lifestyle work doesn’t necessarily have to mean something new*,* but rather to highlight and strengthen*,* clarify and make visible what we already do. (IF 1)*

### Normative restructuring

The health-promoting practice required less time and worked better than expected beforehand, despite the bumpy road due to initial resistance among PHC professionals. This practice developed the professionals’ skills, leading to new insights about the time and commitment required to work health-promotively. An example was that addressing lifestyle habits could start a change process in patients; for example, when they became aware of harmful use of alcohol. Telephone availability at the PHC was described as not being affected to the anticipated extent. During the implementation intervention, the PHC managers had seen the PHC professionals change their attitudes towards health-promotion practice, manifesting in terms of more spontaneous talk about health promotion with a new and more positive tone of dialogue. The PHC professionals had gained their own experiences of health promotion, and participated in discussions out of shared experiences.

*I feel that the conversation [the talk in the workplace about health promotion] has changed; now it is my colleagues who can lead the conversation based on their experience. (IF 1)*

### Relational restructuring

The implementation resulted in a relational restructuring by making different professionals collaborate and thus gain new insights into each other’s work.

*We’ve gained so much on the side because we’ve done it together; the employees have discovered each other [how they work and their competences]. (Manager 5)*

This increased collaboration from working together shed light on existing skills and knowledge about health-promotion practice. The managers emphasized the importance of reviewing and using the available competences.

### Sustainment

Everyone working at the PHC centres was described as being aware of how to work with health-promotive practice after the implementation intervention, even though not all PHC professionals had participated. However, several PHC professionals recognized health-promotive practice as a new routine after the implementation effort, as illustrated in the following dialogue from a FGD.

*- I will continue because I think it’s so easy and works so well and it’s no extra work. (FGD 2:1)*

*- I think*,* to continue to raise the issue at workplace meetings or at our professional meetings. (FGD 2:2)*

*- Yes exactly. If you’re going to try to continue working and everyone’s going to*,* then you need*,* well*,* the nursing group*,* I think*,* what should they focus on? Just raise the issue of wound patients and tobacco*,* they already do that too*,* but they need concreteness: what should we do here. (FGD 2:1)*

*- And coming from the management too*,* for them to raise at workplace meetings that it’s something that is prioritized*,* that we should continue with this. (FGD 2:2)*

## Discussion

This study describes the experiences of PHC professionals, IFs, and managers working with health promotion after a 12-month implementation intervention. The main findings regarding how the health-promoting practice can be normalized include the role of the PHC managers, group dynamics, and inter-professional collaboration; these are discussed below in relation to the three domains of NPT.

### Implementation context

The PHC centres’ environment was influenced by research and guidelines, together with previous frustration over not be able to prioritize health promotion. A strong interrelationship between levels of awareness and attitudes towards addressing lifestyle habits affected the environment, and served as a foundation for the possibility of adapting the execution of the health-promotive practice.

Previous research has emphasized that managers should take an active role in the implementation process, clarifying the vision and goals of the intervention; this requires managers to have knowledge in leadership for implementation [38]. Our findings indicate that the implementation was facilitated by an active leadership, including seeing the potential in the existing team of professionals and using their competence. The role of a manager can bridge the gap between set goals and what is possible to accomplish, and enable tailored strategies [39]. Therefore, in the Act in Time project, support was provided to the PHC managers according to the Astrakan model of leading change [21].

Several barriers and facilitators for working health-promotively in PHC have been reported previously [16]. The present study showed that external factors such as workload and other work tasks affected all the PHC centres’ ability to work with health promotion, and for this reason there was a request for leadership in how to prioritize. The internal factors differentiated the PHC centres in their ability to find solutions to overcome difficulties. We found differences between the professional groups, but there was not one particular professional group that was described as being negative towards health-promotion practice. Instead, this differed between the PHC centres, presumably affected by the existing group dynamics. These findings about group dynamics highlight the importance of understanding the context in which interventions are implemented, in order to tailor strategies [40].

### Implementation mechanisms

Achieving a health-promotion practice required the PHC centres to find communal strategies to address lifestyle habits and to follow up. The PHC professionals requested a key person to lead the group, like the IF, but wanted this to happen in an inter-professional collaboration with a shared ownership of the implementation intervention. Collaboration means that there should be not only communal understanding of an implementation intervention in the professional groups, but also active participation in its design, in order to overcome different perspectives [41] such as how to best treat a patient or to handle different levels of engagement in a group task.

Previous research has found that inter-professional collaboration has the potential to lead to an effective implementation, but requires education and an organized implementation leadership [42]. Another study found that the ability to influence led to increased motivation and a greater likelihood that the new working method would be sustained [43]. The needs of professionals and the visions that need to be realized must be brought together, and this is only possible if there is trust in the organization and opportunity to receive feedback on the benefits of the intervention [44]. The leadership’s ability to communicate during an intervention greatly influences the attitudes of the employees who will carry out the implementation intervention, as well as the possibility to unite the groups around the actions requested [45, 46]. In the present study, feedback from managers about increased statistical measures led to improved motivation. Managers providing such feedback to the working group has proven to be crucial in demonstrating that the manager is serious about the innovation to be implemented [46].

### Implementation outcomes

Health-promotion practice was described as requiring less time and effort than predicted; previously, the informants had thought that health promotion would increase the workload, which would have been a barrier to implementation. This difference between expectations and reality may be explained by the clinical intervention being described as leading to a more structured way of working, and the fact that it was possible to start a change process in patients by simply addressing their lifestyle habits. These new insights changed the attitudes and increased the interest, making it possible to adapt and develop existing ways of working. The implementation intervention can therefore be seen as a dynamic process that needed to be adjusted depending on what happened during the implementation intervention at the specific workplace, as discovered in previous studies [44, 47]. The implementation effort itself enabled improved group dynamics and inter-professional collaboration that opened the doors for the execution of the health-promotive practice, but might also have paved the way for future implementation efforts in the workplace. Further research is needed on the effects of implementation interventions in the PHC context.

Strengths and limitations.

Qualitative interviews are a flexible and powerful data collection method for gaining a deeper understanding of underlying factors that are otherwise difficult to investigate [48], and were therefore used to gain understanding of the informants’ experiences of working health-promotively. In FGDs, the interaction between participants may generate more information, and it may be more comfortable to talk in a group than in individual interviews, but there is a risk that minority opinions may be muted [48]. Both the interviews and the FGDs used, an interview guide inspired by the NPT framework [28]. Appropriate questions for qualitative interviews are considered to be open, neutral, sensitive, and clear [35]. The research team therefore scrutinized the questions in the interview guide before finally deciding how to phrase them.

The project employed a combination of top-down and bottom-up perspectives, and therefore collected data from various organizational levels including senior managers, managers, staff, and IFs. To understand the behaviour of a working group and the changes that are and are not made, an understanding of both the top-down and bottom-up perspectives is required [49]. In this study, the interviewed senior managers were also part of the projects advisory board, which might have influenced their statements positively. Nevertheless, their interview data were primarily categorized under implementation context. One limitation was that PHC managers or IFs selected participants for the FGDs, but there was a spread in terms of different professions and experiences of primary care. A few participants in the FGDs had to decline participation due to illness, but recommended group sizes were obtained [50]. Men and women were both represented in the interviews and FGDs. Although there was a higher proportion of women, this is representative of the fact that the health care workforce in general is predominantly women. The data collection with 19 interviews and five FGDs resulted in a large amount of data, but this was made manageable by the structured approach to data analysis and the use of NPT for the deductive analysis.

The deductive analysis method with NPT was performed with openness to additional perspectives. NPT provided new insight at a deeper level, and brought even more structure to the data. This resulted in some codes being categorized into other groups than in the inductive phase of the analysis process. The domain of implementation outcome included statements from PHC professionals experiencing integration of the health promotion into practice, thereby providing a positive finding.

In the present study, all 12 NPT constructs were used to analyse the data. To our knowledge, this is a new approach, as previous work has primarily explored the constructs relating to implementation mechanisms, and has struggled to separate the constructs from each other [51]. The lack of previous studies using the constructs of implementation context and implementation outcome reduced the opportunity for comparing the findings with other studies. The findings enhance understanding of PHC professionals´ experiences of increasing their health promotion practice, and can be useful when planning for scaling and facilitate adoption across other PHC contexts. Further research addressing all the constructs of the NPT is needed to fully evaluate the potential of using NPT as an implementation framework, and to understand not only implementation mechanism but also implementation context and outcome.

## Conclusions

Health-promotion practice can be normalized as routine PHC work with targeted support, but requires tailored strategies in the workplace that rely on existing group dynamics and the manager’s role. To create motivation for working health-promotively, inter-professional collaboration is a key factor that ensures shared ownership of the implementation intervention.

## Supplementary Information

Supplementary Material 1.

Supplementary Material 2.

Supplementary Material 3.

## Data Availability

The data generated and analysed during the current study are not publicly available because they contain information that could compromise the integrity of the participants. They are available in Swedish from the corresponding author at reasonable request.

## Abbreviations

EF: external facilitator
FGD: focus group discussion
IF: internal facilitator
NPT: normalization process theory
PHC: primary health care
