# Supplementary material for: Act in time: primary health care professionals’, internal facilitators’, and managers’ experiences of working health-promotively after a 12-month implementation intervention: a qualitative study using normalization process theory
Source: BMC Prim Care. 2026 Jan 22;27:31. doi: 10.1186/s12875-026-03181-0 (PMC12857077; doi:10.1186/s12875-026-03181-0)
Supplement: Supplementary file 1 — Supplementary Material 1. [file 12875_2026_3181_MOESM1_ESM.pdf]

Region Örebro län

Engelska

You can make a difference to your health by adopting healthy habits. The lifestyle choices that are most likely to cause disease/ill health are: using tobacco, drinking too much alcohol, not getting enough exercise, and unhealthy eating habits. It’s helpful if you complete this questionnaire prior to your appointment. That way, healthcare staff can work together with you in a personalised way to help you achieve the best health possible.

Tobacco (Tobak)

1. My smoking habits
   - ☐ I’ve never smoked
   - ☐ I quit smoking more than 6 months ago
   - ☐ I quit smoking less than 6 months ago
   - ☐ I smoke, but not every day
   - ☐ I smoke every day:
     - ☐ 1–9 cigarettes per day
     - ☐ 10–19 cigarettes per day
     - ☐ 20 or more cigarettes per day

2. My *snus* (smokeless tobacco) habits
   - ☐ I’ve never used *snus*
   - ☐ I quit using *snus* more than 6 months ago
   - ☐ I quit using *snus* less than 6 months ago
   - ☐ I use *snus*, but not every day
   - ☐ I use *snus* every day. I use:
     - ☐ 1–3 cans per week
     - ☐ 4–6 cans per week
     - ☐ 7 or more cans per week.

Alcohol (Alkohol)

A ‘standard glass’ means:

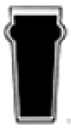	**500 ml low-alcohol beer (*folköl*)**	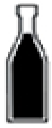	**330 ml strong beer (*starköl*)**	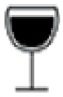	**120–150 ml white or red wine**	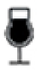	**80 ml fortified wine (*starkvin*)**	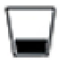	**40 ml spirits, e.g. whisky**

1. How many standard glasses of alcohol do you drink in an ordinary week?
   - ☐ I don’t drink alcohol
   - ☐ Less than 1 standard glass per week
   - ☐ 1–4 standard glasses per week
   - ☐ 5–9 standard glasses per week
   - ☐ 10–14 standard glasses per week
   - ☐ 15 or more standard glasses per week

2. If you’re female, how often do you drink 4 standard glasses or more on the same occasion?
   If you’re male, how often do you drink 5 standard glasses or more on the same occasion?
   - ☐ Never
   - ☐ Less than once a month
   - ☐ Every month
   - ☐ Every week
   - ☐ Every day or almost every day

Utvecklingsenheten, HS Verksamhets- och ledningsstöd 2023

Physical activity (Fysisk aktivitet)

1. In an ordinary week, how much time do you spend on physical exercise that causes you to become short of breath, e.g. running, fitness exercises (*gympa*), or ball sports?
   - ☐ Zero
   - ☐ Less than 30 minutes
   - ☐ 30–60 minutes (0.5–1 hour)
   - ☐ 60–90 minutes (1–1.5 hours)
   - ☐ 90–120 minutes (1.5–2 hours)
   - ☐ More than 120 minutes (2 hours or more)

2. In an ordinary week, how much time do you spend on incidental exercise, e.g. walks, cycling, or gardening? Add up all the time you spend exercising.
   - ☐ Zero
   - ☐ Less than 30 minutes
   - ☐ 30–60 minutes (0.5–1 hour)
   - ☐ 60–90 minutes (1–1.5 hours)
   - ☐ 90–150 minutes (1.5–2.5 hours)
   - ☐ 150–300 minutes (2.5–5 hours)
   - ☐ More than 300 minutes (5 hours or more)

Sedentary time (Stillasittande)

How much time do you spend **sitting** during an ordinary day?
- ☐ Basically the whole day
- ☐ 13–15 hours
- ☐ 10–12 hours
- ☐ 7–9 hours
- ☐ 4–6 hours
- ☐ 1–3 hours
- ☐ Zero hours

Food (Mat)

1. How often do you eat vegetables and/or root vegetables (fresh, frozen, or pre-prepared)?
   - ☐ Twice a day or more
   - ☐ Once a day
   - ☐ A few times a week
   - ☐ Once a week or less often

2. How often do you eat fruit and/or berries (fresh, frozen etc.)?
   - ☐ Twice a day or more
   - ☐ Once a day
   - ☐ A few times a week
   - ☐ Once a week or less often

3. How often do you eat fish or shellfish as a main dish, in a salad, or as a sandwich topping?
   - ☐ Three times a week or more
   - ☐ Twice a week
   - ☐ Once a week
   - ☐ A few times a month or less often

4. How often do you eat cakes/buns/biscuits, chocolate/sweets, or potato crisps, or drink soft drinks/sweetened fruit drinks (*saft*)?
   - ☐ Twice a day or more
   - ☐ Once a day
   - ☐ A few times a week
   - ☐ Once a week or less often

5. How often do you eat breakfast?
   - ☐ Every day
   - ☐ Almost every day
   - ☐ A few times a week
   - ☐ Once a week or less often
