# Supplementary material for: Act in time: primary health care professionals’, internal facilitators’, and managers’ experiences of working health-promotively after a 12-month implementation intervention: a qualitative study using normalization process theory
Source: BMC Prim Care. 2026 Jan 22;27:31. doi: 10.1186/s12875-026-03181-0 (PMC12857077; doi:10.1186/s12875-026-03181-0)
Supplement: Supplementary file 2 — Supplementary Material 2. [file 12875_2026_3181_MOESM2_ESM.docx]

## Normalization Process Theory (NPT) coding map and definitions:

| ***NPT domains***  **Implementation contexts**  Contexts are patterns of social relations and structures that unfold over time and across settings. They make up the implementation environment**.** |  | | |  |
| --- | --- | --- | --- | --- |
|  | ***Constructs*** | ***Definition*** | ***Subconstruct*** | **Keywords** |
|  | **Strategic intentions** | How do contexts shape the formulation and planning of interventions and their components? |  | Research and development  Governance  Policy and politics  Current social situation |
|  | **Adaptive execution** | How do contexts affect the ways in which users can find and enact workarounds that make an intervention and its components a workable proposition in practice? |  | The relationship between manager and employee  Pre-conditions  Resources  Group size |
|  | **Negotiating capability** | How do contexts affect the extent that an intervention and its components can fit, or be integrated, into existing ways of working by their users? |  | Priorities  Administrative barriers / opportunities  Relevance  Fear of intrusion |
|  | **Reframing organisational logic** | How do existing social structural and social cognitive resources shape the implementation environment? |  | Attitudes  Culture  Group dynamics |
| **Implementation mechanisms**  Mechanisms are revealed through purposive social action—collaborative work—that involves the investment of personal and group resources to achieve goals. | **Coherence-building**  (What is it?) | How do people work together in everyday settings to understand and plan the activities that need to be accomplished to put an intervention and its components into practice? | **Differentiation:** How do people distinguish interventions and their components from their current ways of working?  **Communal specification:** How do people collectively agree about the purpose of interventions and their components?  **Individual specification:** How do people individually understand what interventions and their components require of them?  **Internalization:** How do people construct the potential value of interventions and their components for their work? |  |
|  | **Cognitive participation**  (Who does it?) | How do people work together to create networks of participation and communities of practice around interventions and their components? | **Initiation:** How do key individuals drive interventions and their components forward?  **Enrolment:** How do people join in with interventions and their components?  **Legitimation:** How do people agree that interventions and their components are the right thing to do and should be part of their work?  **Activation:** How do people continue to support interventions and their components? |  |
|  | **Collective action**  (How does it get done?) | How do people work together to enact interventions and their components? | **Interactional workability:** How do people do the work required by interventions and their components?  **Relational integration:** How does using interventions and their components affect the confidence that people have in each other?  **Skillset workability:** How is the work of interventions and their components appropriately allocated to people?  **Contextual integration:** How is the work of interventions and their components supported by host organizations? |  |
|  | **Reflexive monitoring**  (Why did it happen?) | How do people work together to appraise interventions and their components? | **Systematisation:** How do people access information about the effects of interventions and their components?  **Communal appraisal:** How do people collectively assess interventions and their components as worthwhile?  **Individual appraisal:** How do people individually assess interventions and their components as worthwhile?  **Reconfiguration:** How do people modify their work in response to their appraisal of interventions and their components? |  |
| **Implementation outcomes**  The practical effects of implementation mechanisms at work (i.e. what is visible in terms of how things change as implementation processes proceed). | **Intervention performance** | What practices have changed as the result of interventions and their components being operationalized, enacted, and reproduced over time and across settings? |  | Increased focus  Holistic perspective  More structure  Involving patients |
|  | **Normative restructuring** | How has working with interventions and their components changed the norms, rules, and resources that govern action? |  | Skills enhancement  New insights  Culture  Increased awareness |
|  | **Relational restructuring** | How has working with interventions and their components changed the ways people are organized and relate to each other? |  | Participation  Making resources visible  Frustration |
|  | **Sustainment** | How have interventions and their components become incorporated in practice? |  | Normalization  Routine |
