# Supplementary material for: Act in time: primary health care professionals’, internal facilitators’, and managers’ experiences of working health-promotively after a 12-month implementation intervention: a qualitative study using normalization process theory
Source: BMC Prim Care. 2026 Jan 22;27:31. doi: 10.1186/s12875-026-03181-0 (PMC12857077; doi:10.1186/s12875-026-03181-0)
Supplement: Supplementary file 3 — Supplementary Material 3. [file 12875_2026_3181_MOESM3_ESM.docx]

| **Managers** | **PHC professionals** | **Internal facilitators** |
| --- | --- | --- |
| What are your thoughts about the clinical process (screening forms, giving advice/counselling, documentation)? Benefits and drawbacks? | | |
| How would you describe the integration of the health-promotive way of working in clinical practice? What does it look like? Give examples.  Is everyone on board? | | |
| Would you say that there is a mutual understanding between colleagues and between different professions regarding health-promotive work?  Elaborate; how is this visible? | | |
| What do you think will be of importance for sustainability?  Challenges and solutions  What would be your role? | | |
| What advice would you give to a primary healthcare centre that wants to implement a more health-promotive way of working – what would your advice be and to whom would you direct the advice? | | |
| Can you please tell me about the change journey; how has the health-promotive way of working been implemented at the primary healthcare centre?  Facilitating factors and barriers  Describe your role in the implementation process  Challenges, solutions, and support | | Can you please describe how you prepared for the role of IF? |
|  | | How would you describe the implementation intervention?  What worked/did not work and why? |
|  | What are your experiences from the implementation support?  Facilitation, support, activities  Participation in the change | Describe the activities that you have done as an IF. What was most impactful/important?  Challenges and workarounds  Facilitating factors and support  Competing interests |
|  | Does the health-promotive way of working fit the clinical work? |  |
|  | Describe whether you received feedback. If so: from whom, how, was it helpful? |  |
|  | Have you identified new groups of patients that you now address lifestyle habits with? |  |
|  | Describe how the patients react |  |

IF = internal facilitator, PHC = primary health care
